# Cystitis Due to Bacillus Coli Communis

**Published:** 1908-08-01

**Authors:** 


### CYSTITIS DUE TO BACILLUS COLI COMMUNIS.

### THE VALUE OF COLI-VACCINES.

Many different bacteria may give rise to cystitis. Amongst these staphylococci, streptococci, gonococci, tubercle bacilli, and even diphtheria bacilli and the bacillus lactis aërogenes have been found. In a large proportion of cases, however, the bacillus coli communis is the sole causal organism.

This is a very important point from a therapeutic standpoint, for it has become evident that, of all vaccine treatments, that by coli-vaccine for urinary infection by the colon bacillus is followed by some of the very best results. The doses used are up to the equivalent of 500 millions of the bacteria, given hypodermically. The bulk of the dose is about ten drops, so that the injection causes little discomfort. The doses are repeated at intervals of from ten days to a fortnight. Improvement is well advanced after the second dose, and, although a third dose is sometimes required, there are many cases in which there may be doubt as to whether it is really necessary. The most severe cases may require more than three doses, but this is unusual.

It is true that mild infections are rapidly cured by the administration of ammonium benzoate or methylene compounds, and it is unnecessary to employ the vaccine if the cystitis or pyelitis rapidly gives way after the exhibition of these. There are, however, many other cases in which antipyuric remedies given by the mouth are very slow in bringing about the desired result. This applies, for example, in some instances of pyelo-nephritis associated with pregnancy, to cystitis in women who are not pregnant, to men with cystitis from enlargement of the prostate or from stricture of the urethra, and to cases of cystitis in patients suffering from locomotor ataxia, spastic paraplegia, transverse myelitis, and the like, with consequent retention of urine and overflow. It is in such cases as these that the coli-vaccine treatment is often of immense benefit, provided that the organism causing the cystitis or nephritis is proved by bacteriological cultivations of the urine to be the bacillus coli communis.

We have pointed out elsewhere that antipyuric drugs are only able to cure pyuria when there is no breach of surface in the urinary tracts; that is to say, they are not able to kill bacteria which have found a foothold beneath the surface of the bladder or other part of the passages through which the urine flows; they are only able to inhibit the rate of multiplication of the bacteria in the urine itself, and it is only when the rate of multiplication is thus made less than the rate at which the organisms are evacuated by micturition that the antipyuric remedies bring about a cure. The urine cannot be made so antiseptic that the bacteria in it become killed; it can only be rendered a less good medium for the growth of the bacteria than ordinary urine is; and it is only the bacteria in the urine itself—not those in the walls of the urinary passages—whose rate of multiplication can thus be inhibited.

The coli-vaccine, on the other hand, seems to render the patient's tissues more active than before in the destruction of the bacteria which are lodged in them. The vaccine produces the cure in quite a different way to that in which urotropine, for example, acts. There is no reason why both antipyuric remedies and vaccine injections should not be used simultaneously; indeed, it is wise that they should be so used. The vaccines, however, are able to cure cases in which drugs administered by the mouth fail, and this is a point of extreme importance in practice. In any case of severe pyuria, therefore, it is well worth while to have bacteriological investigations of the urine made by cultural methods; if, as is often the case, bacillus coli communis is present in pure, or almost pure, growth, a vaccine can be prepared from the patient's own organisms, and relief is to be expected from a comparatively small number of injections of the vaccine so prepared.

